# Supplementary material for: Brachiopod and mollusc biomineralisation is a conserved process that was lost in the phoronid–bryozoan stem lineage
Source: EvoDevo. 2022 Sep 19;13:17. doi: 10.1186/s13227-022-00202-8 (PMC9484238; doi:10.1186/s13227-022-00202-8)
Supplement: Supplementary file 1 — Additional file 1: Figure S1. Phylogenetic relationships of the homeodomain transcription factor proteins engrailed, goosecoid and distal-less in various metazoan taxa. Blue font denotes lophophorates. SH-like support values are indicated for select nodes. Figure S2. Phylogenetic relationships of the signalling molecule BMP2–4 to closely related proteins in various metazoan taxa. Blue font denotes lophophorates. SH-like support values are indicated for select nodes. Figure S3. Phylogenetic relationships of ferritins from various metazoan taxa to fungal orthologues. Blue font denotes lophophorates. SH-like support values are indicated for select nodes. Figure S4. Phylogenetic relationships of calmodulin to other calcium-binding proteins in various metazoan taxa. Blue font denotes lophophorates. SH-like support values are indicated for select nodes. Figure S5. Phylogenetic relationships of perlucin orthologues to other lectin-related proteins in various metazoan taxa. Blue font denotes lophophorates. SH-like support values are indicated for select nodes. Figure S6. Phylogenetic relationships of mpox (mantle peroxidases) to other peroxidase-related proteins in various metazoan taxa. Blue font denotes lophophorates. SH-like support values are indicated for select nodes. Figure S7. Alignment of the SP1 and SP2 protein sequences from four brachiopod species. Table S1. Reference sequences used to search genomes/transcriptomes for each gene of interest [file 13227_2022_202_MOESM1_ESM.docx]

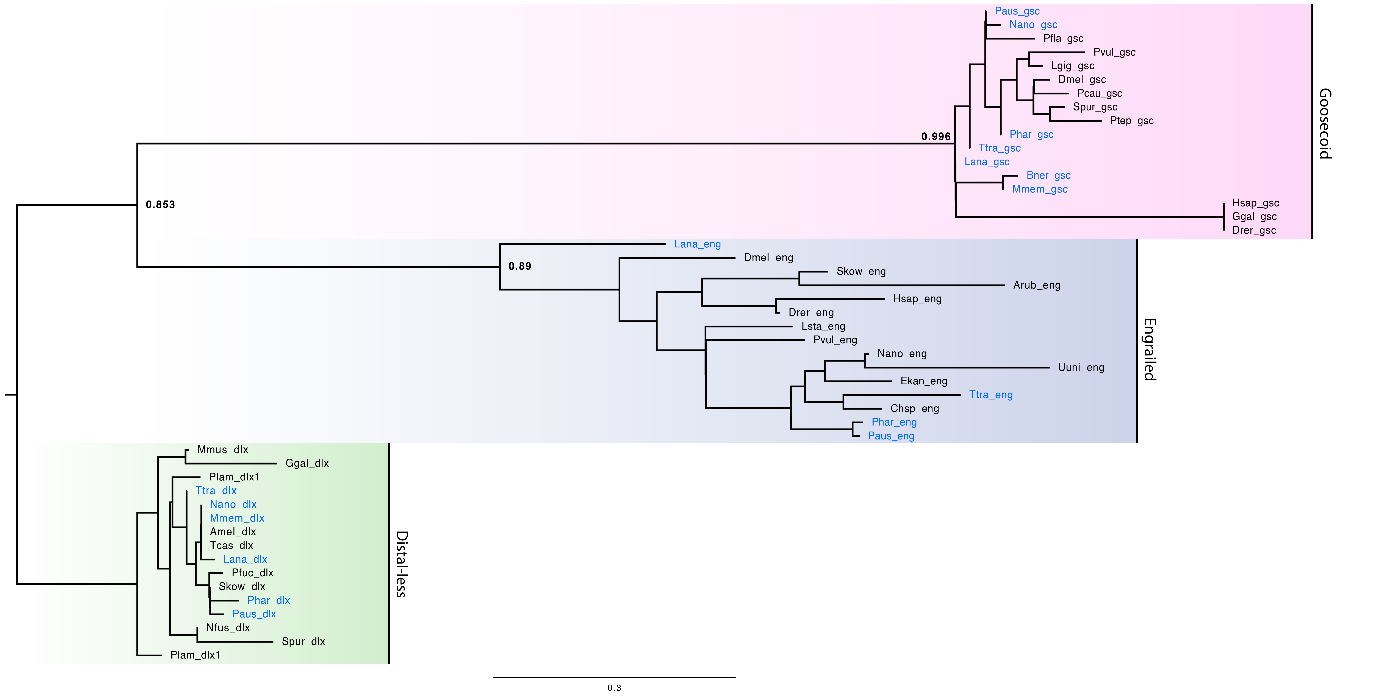


**Additional figure 1.** Phylogenetic relationships of the homeodomain transcription factor proteins engrailed, goosecoid and distal-less in various metazoan taxa. Blue font denotes lophophorates. SH-like support values are indicated for select nodes.


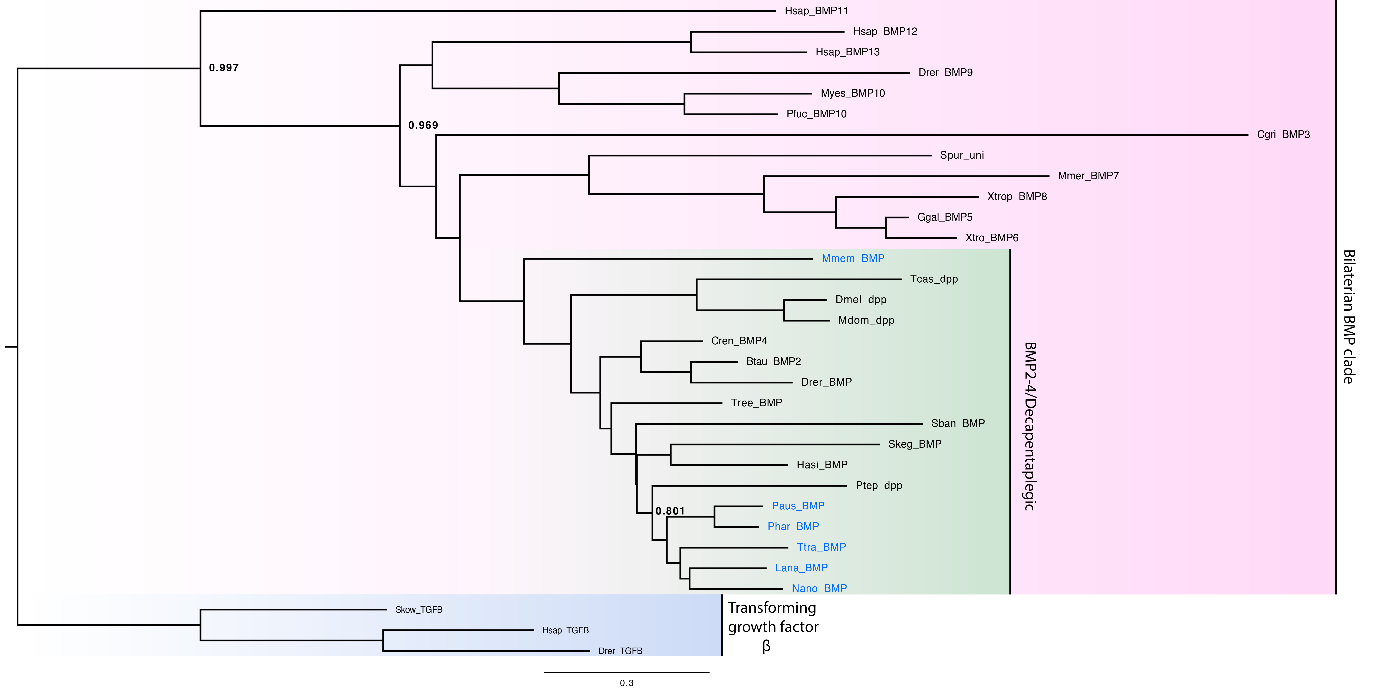


**Additional figure 2.** Phylogenetic relationships of the signalling molecule BMP2-4 to closely related proteins in various metazoan taxa. Blue font denotes lophophorates. SH-like support values are indicated for select nodes.


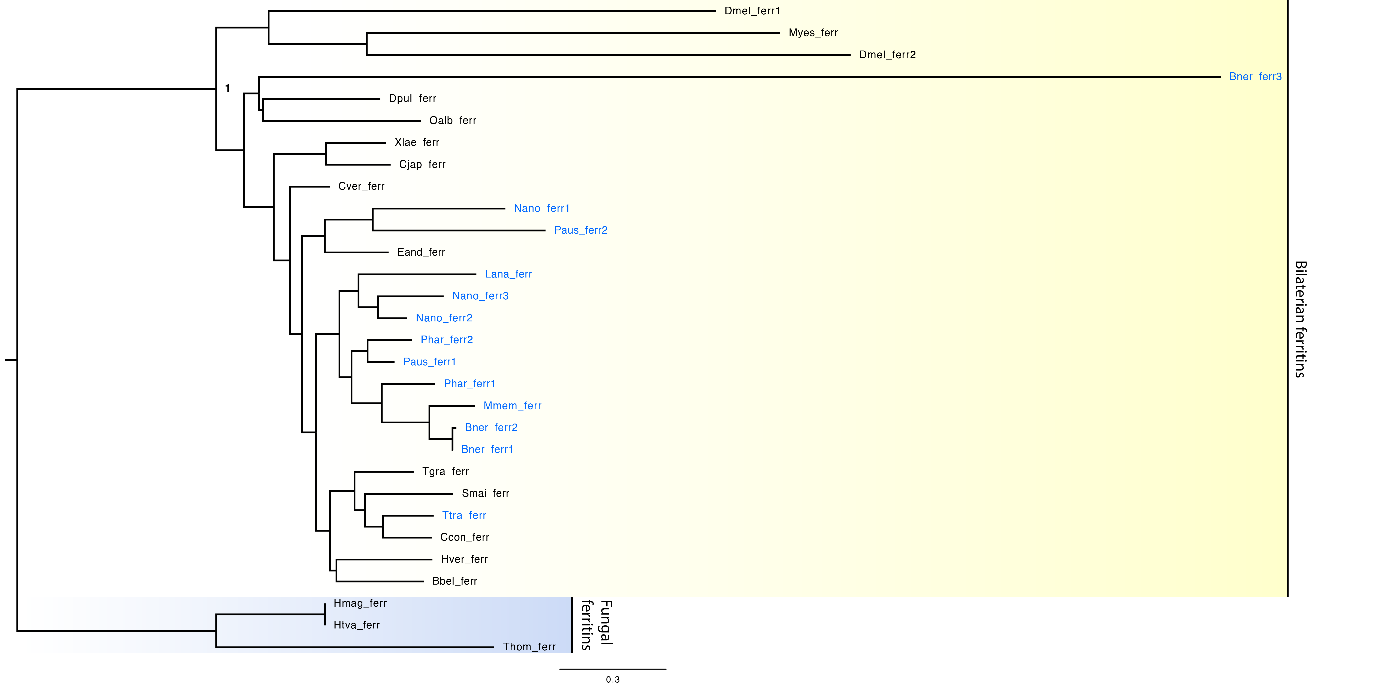


**Additional figure 3.** Phylogenetic relationships of ferritins from various metazoan taxa to fungal orthologues. Blue font denotes lophophorates. SH-like support values are indicated for select nodes.


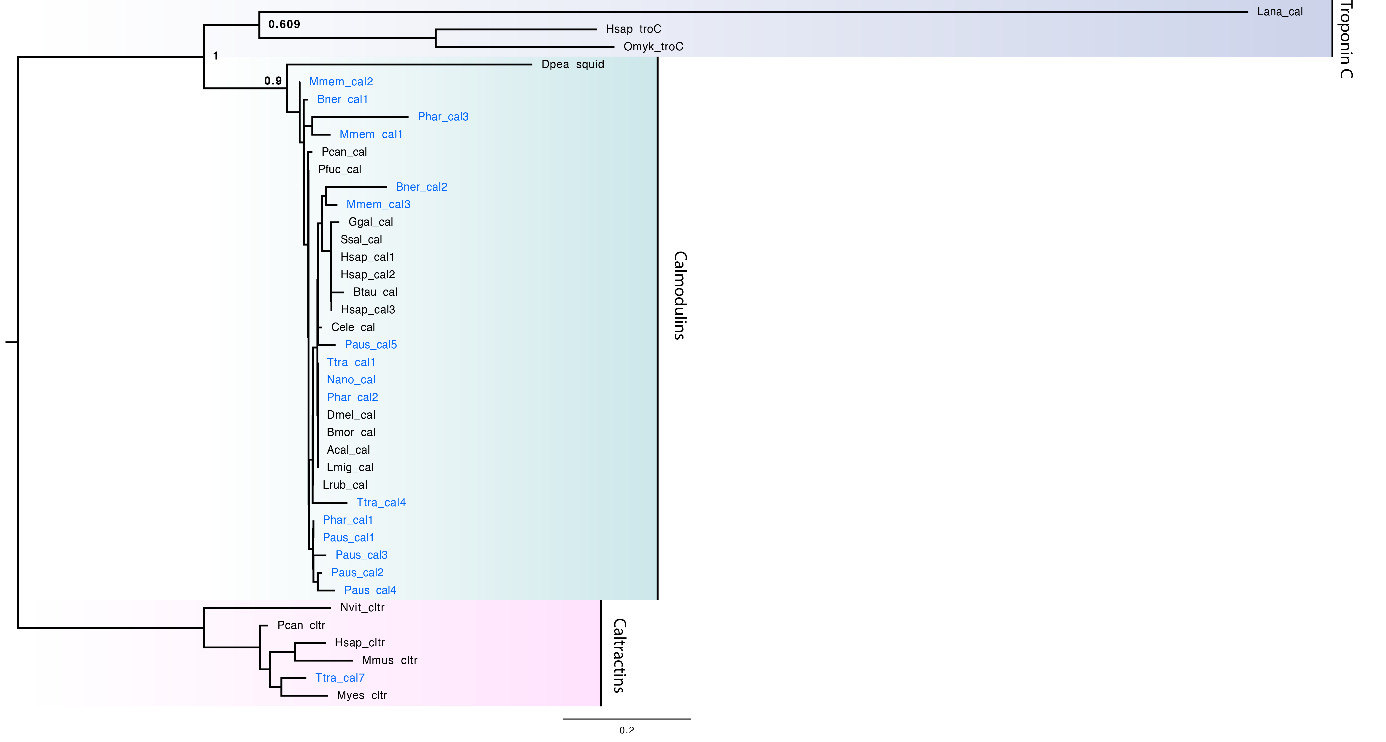


**Additional figure 4.** Phylogenetic relationships of calmodulin to other calcium-binding proteins in various metazoan taxa. Blue font denotes lophophorates. SH-like support values are indicated for select nodes.


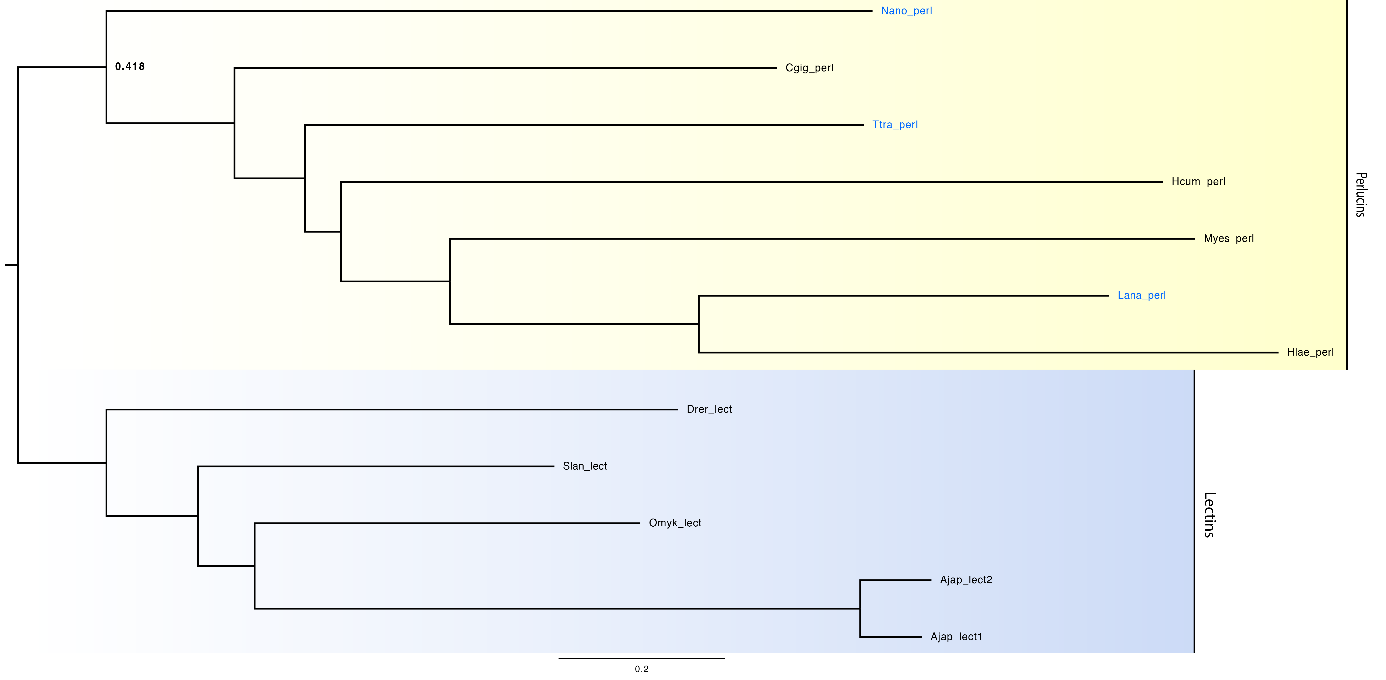


**Additional figure 5.** Phylogenetic relationships of perlucin orthologues to other lectin-related proteins in various metazoan taxa. Blue font denotes lophophorates. SH-like support values are indicated for select nodes.


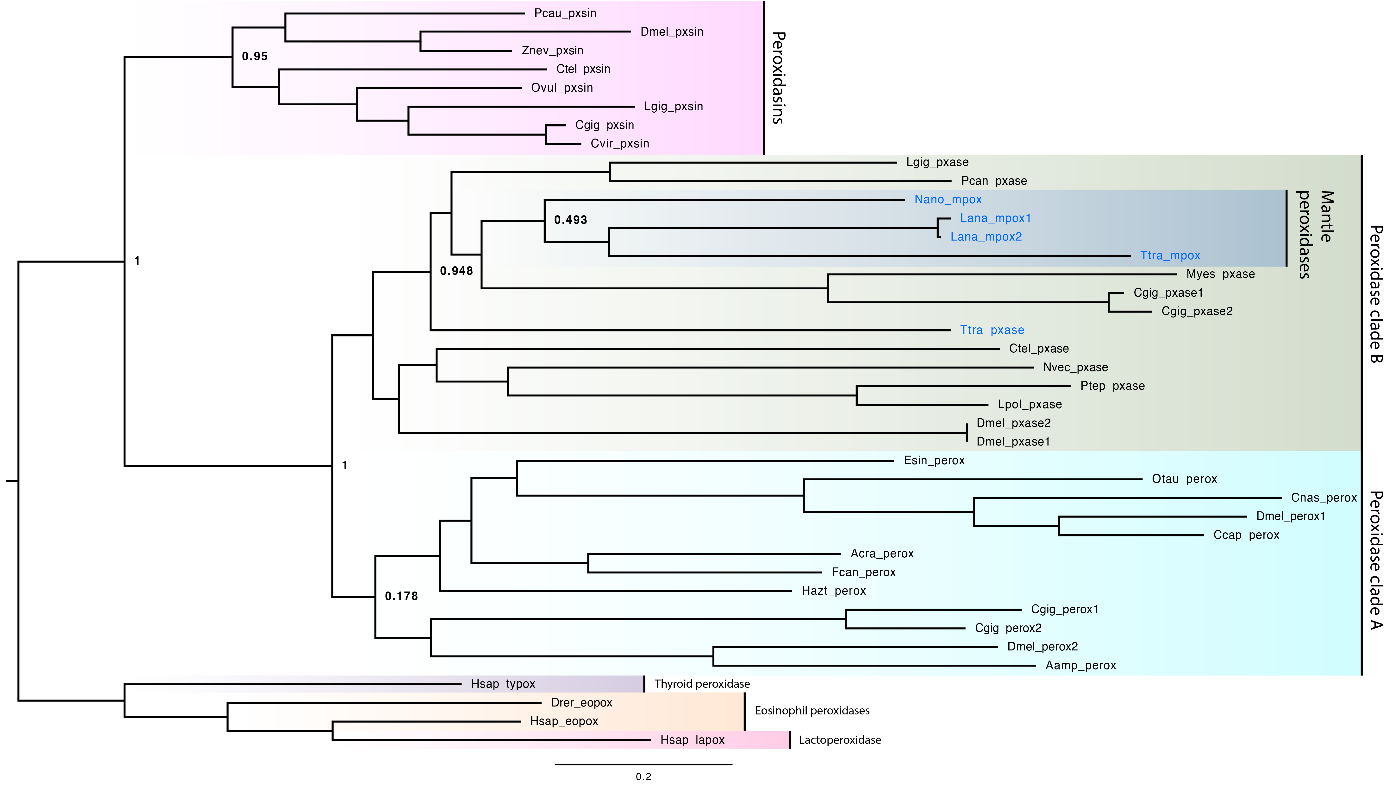
 **Additional figure 6.** Phylogenetic relationships of mpox (mantle peroxidases) to other peroxidase-related proteins in various metazoan taxa. Blue font denotes lophophorates. SH-like support values are indicated for select nodes.

**Additional figure 7.** Alignment of the *SP1* and *SP2* protein sequences from four brachiopod species.

**Additional table 1.** Reference sequences used to search genomes/transcriptomes for each gene of interest.

| **Gene name** | **Genbank accession** | **Organism** | **Notes** |
| --- | --- | --- | --- |
| Engrailed | ALS19757.1 | *Terebratalia transversa* |  |
| Distal-less | BBB21813.1 | *Lingula anatina* |  |
| Decapentaplegic/BMP2-4 | XP_013396300.1 | *Lingula anatina* |  |
| Goosecoid | XP_013379052 | *Lingula anatina* |  |
| Mpox (mantle peroxidase) | BBB21814.1 | *Lingula anatina* |  |
| Ferritin | XP_023933523 | *Lingula anatina* |  |
| Calmodulin | NP_001191509.1 | *Aplysia californica* |  |
| Perlucin | AGI61062.1 | *Hyriopsis cumingii* |  |
| F10023803 (SP1) |  | *Magellania venosa* | From Jackson et al. 2015 |
| R20087389 (SP2) |  | *Magellania venosa* | From Jackson et al. 2015 |
